# Roles of FGFs As Paracrine or Endocrine Signals in Liver Development, Health, and Disease

**DOI:** 10.3389/fcell.2016.00030

**Published:** 2016-04-13

**Authors:** Nobuyuki Itoh, Yoshiaki Nakayama, Morichika Konishi

**Affiliations:** ^1^Medical Innovation Center, Kyoto University Graduate School of MedicineKyoto, Japan; ^2^Department of Microbial Chemistry, Kobe Pharmaceutical UniversityKobe, Japan

**Keywords:** carcinoma, development, disease, FGF, liver, metabolism

## Abstract

The liver plays important roles in multiple processes including metabolism, the immune system, and detoxification and also has a unique capacity for regeneration. FGFs are growth factors that have diverse functions in development, health, and disease. The FGF family now comprises 22 members. Several FGFs have been shown to play roles as paracrine signals in liver development, health, and disease. FGF8 and FGF10 are involved in embryonic liver development, FGF7 and FGF9 in repair in response to liver injury, and FGF5, FGF8, FGF9, FGF17, and FGF18 in the development and progression of hepatocellular carcinoma. In contrast, FGF15/19 and FGF21 are endocrine signals. FGF15/19, which is produced in the ileum, is a negative regulator of bile acid metabolism and a stimulator of gallbladder filling. FGF15/19 is a postprandial, insulin-independent activator of hepatic protein and glycogen synthesis. It is also required for hepatocellular carcinoma and liver regeneration. FGF21 is a hepatokine produced in the liver. FGF21 regulates glucose and lipid metabolism in white adipose tissue. Serum FGF21 levels are elevated in non-alcoholic fatty liver. FGF21 also protects against non-alcoholic fatty liver. These findings provide new insights into the roles of FGFs in the liver and potential therapeutic strategies for hepatic disorders.

## Introduction

The prototypic fibroblast growth factors (FGFs), FGF1 and FGF2, which were originally isolated from the brain as growth factors for fibroblasts, are multi-functional signaling proteins of ~150 amino acids that exert diverse activities in cell proliferation, angiogenesis, neuronal cell growth and survival, and wound healing with widespread expression profiles in embryos and adults (Burgess and Maciag, 1989; Baird and Klagsbrun, 1991). The FGF family now comprises 22 members including FGF1–FGF23 in humans and mice. These FGFs also have diverse functions in development, health, and disease. The human and mouse FGF families do not include FGF15 or FGF19, respectively, because they are orthologs. Although these orthologs have been named FGF15 in rodents and FGF19 in other vertebrates, they are typically referred to as FGF15/19 (Goetz and Mohammadi, 2013; Ornitz and Itoh, 2015).

The liver, which is the largest organ in the body, plays important roles in multiple processes including catabolism and anabolism, the immune system, and detoxification. The liver also has a unique capacity for regeneration with the potential for the full restoration of liver mass and function even following massive damage (Taub, 2004; Bhatia et al., 2014). Several FGFs function as paracrine or endocrine signals in liver development, health, and disease. These findings provide new insights into the roles of FGFs in the liver and potential therapeutic strategies for hepatic disorders. A succinct review of the roles of FGFs in the liver is provided herein.

## The FGF family

**Figure 1:**
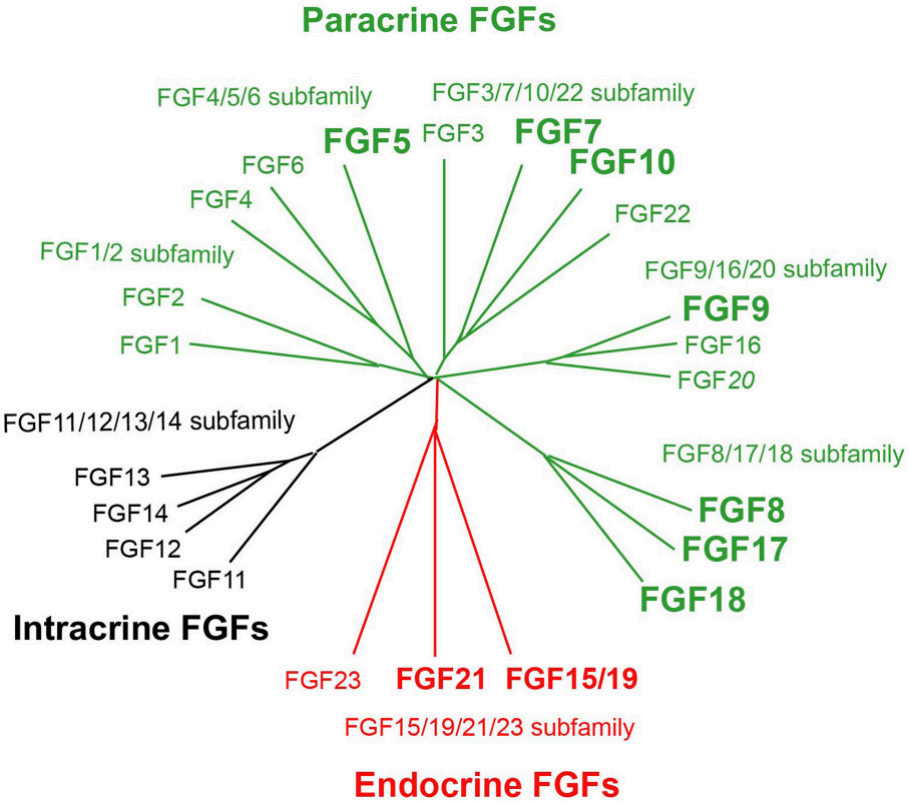
**Evolutionary relationships within the human FGF family**. Phylogenetic analyses suggest that 22 members of the FGF family are classified into seven subfamilies including the FGF1/2, FGF3/7/10/22, FGF4/5/6, FGF8/17/18, FGF9/16/20, FGF11/12/13/14, and FGF15/19/21/23 subfamilies. Branch lengths are proportional to the evolutionary distance between each FGF. FGFs are also classified into paracrine, endocrine, and intracrine FGFs based on their mechanisms of action. Of these, FGF5, FGF7, FGF8, FGF9, FGF10, FGF17, FGF18, FGF15/19, and FGF21 play roles in liver development, health, and disease.

The FGF family includes 22 FGF proteins of ~150–300 amino acids with a conserved core (~30–60% amino acid identity) of ~120 amino acids. Phylogenetic analyses of the FGF family have revealed potential evolutionary relationships with seven subfamilies in this family. FGFs have been classified into paracrine, endocrine, and intracrine FGFs based on their mechanisms of action (Figure 1). Mice lacking these FGFs indicate their crucial roles in development and health. Paracrine FGFs, which comprise 15 members, are locally secreted signals that mainly function in multiple developmental and physiological processes. Endocrine FGFs, which comprise three members, are secreted endocrine signals that mainly function in multiple metabolic processes. *FGF* gene variations in humans also result in various diseases. These findings indicate that paracrine and endocrine FGFs are crucial for ensuring proper development and health in mice and humans. In contrast, intracrine FGFs, which comprise four members, are not secreted signals that play roles in the regulation of electrical excitability in neurons in an intracrine manner (Goetz and Mohammadi, 2013; Ornitz and Itoh, 2015). Several paracrine and endocrine FGFs, but not intracrine FGFs are involved in liver development, health, and disease as described below.

## Roles of paracrine FGFs in the liver

**Figure 2:**
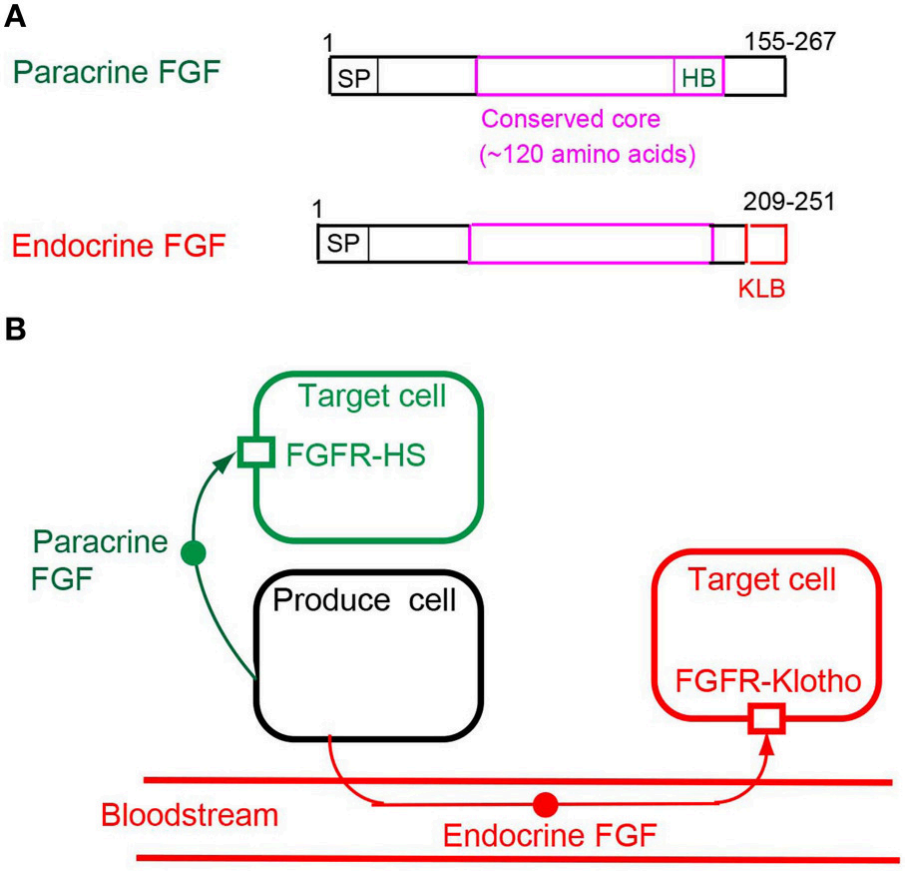
**(A)** Schematic representations of paracrine and endocrine FGF structures. SP, HB, and KLB indicate a secreted signal sequence, heparan sulfate-binding site, and Klotho-binding site, respectively. **(B)** Mechanisms of action of paracrine and endocrine FGFs. Paracrine FGFs are locally secreted signals that act on nearby target cells by diffusion, with functions in multiple developmental and physiological processes. Endocrine FGFs are secreted endocrine signals that act on distant target cells through the bloodstream, with functions in multiple metabolic processes. FGFR-HS and FGFR-Klotho indicate the FGFR-heparan sulfate complex and FGFR-Klotho complex, respectively.

**Figure 3:**
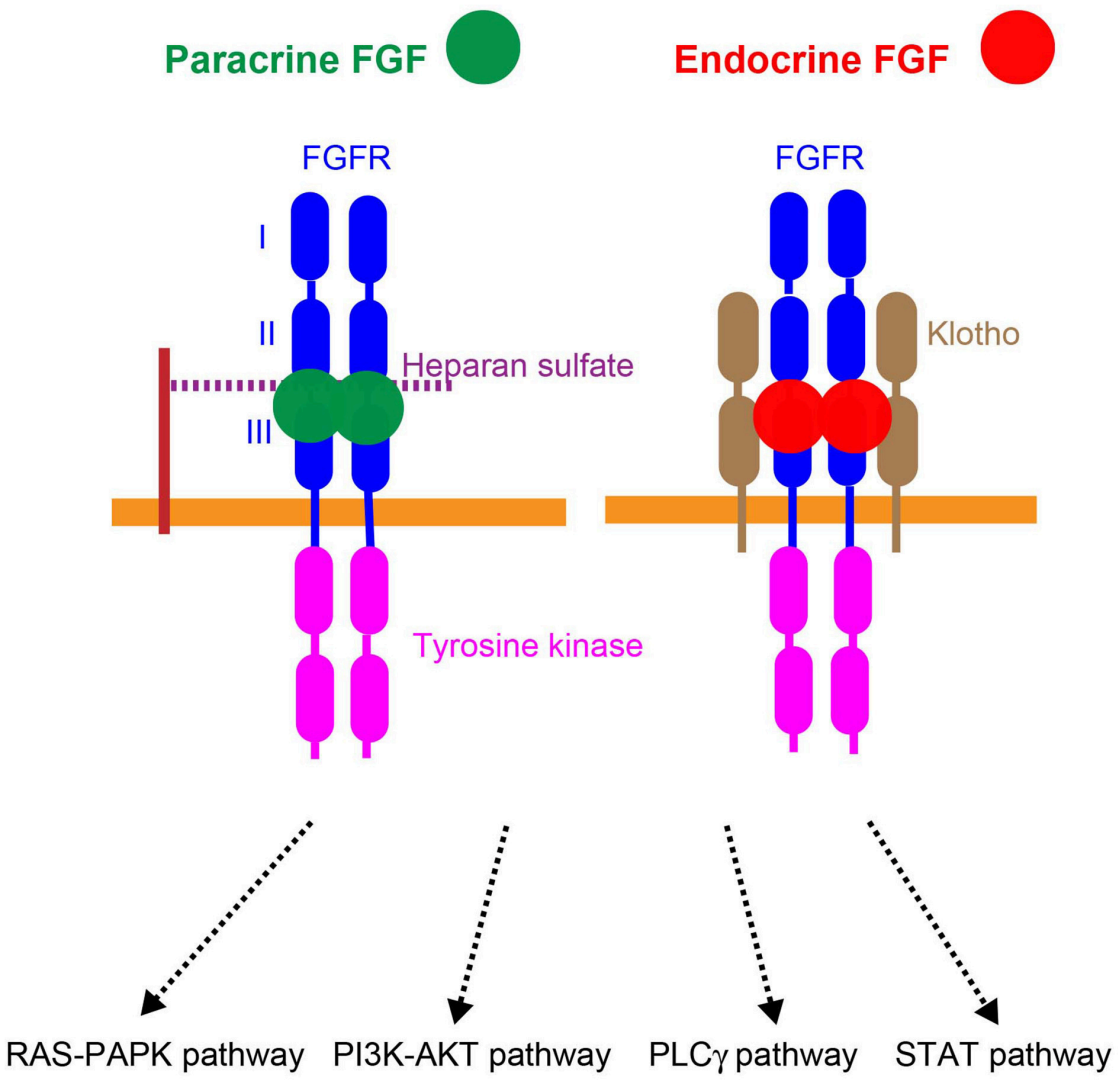
**Mechanisms of action of paracrine and endocrine FGFs**. Paracrine FGFs specifically bind to the FGFR-heparan sulfate complex and activate FGFR tyrosine kinase. This activation, in turn, induces the activation of the RAS–MAPK, PI3K–AKT, PLCγ, and STAT pathways. Endocrine FGFs specifically bind to the FGFR-Klotho complex and activate tyrosine kinase. This activation, in turn, induces the activation of intracellular pathways.

Paracrine FGFs, which comprise 15 members including FGF1-FGF6, FGF7-FGF10, FGF16-FGF18, FGF20, and FGF22, have a secreted signal sequence and heparan sulfate-binding site at their amino and carboxyl termini, respectively (Figures 1, 2A). Paracrine FGFs act on nearby target cells as locally secreted signals via diffusion. Heparan sulfate chains, which are long linear carbohydrate chains of repeating sulfated glucuronic acid linked to N-acetylglucosamine disaccharides, are covalently linked to specific cell surface transmembrane-type proteins. Heparan sulfate functions to sequester FGFs and modulate their diffusion. This modulation of diffusion directs paracrine FGFs as local signals (Figures 2B, 3; Goetz and Mohammadi, 2013; Ornitz and Itoh, 2015).

Paracrine FGFs mediate biological responses by binding to cell surface FGF receptors (FGFRs) with heparan sulfate as a co-factor. Seven major FGFR proteins (FGFRs 1b, 1c, 2b, 2c, 3b, 3c, and 4) with differing ligand-binding specificities are generated from the *FGFR1, FGFR2, FGFR3*, and *FGFR4* genes by alternative splicing. Heparan sulfate is necessary for stable interactions with FGFRs, and also independently interacts with FGFs and FGFRs. The FGF-FGFR-heparan sulfate complex leads to FGFR dimerization, which directs the activation of FGFR intracellular tyrosine kinase domains, followed by that of key intracellular signaling pathways including the RAS-mitogen-activated protein kinase (MAPK), phosphoinositide 3-kinase (PI3K)-AKT, phospholipase Cγ (PLCγ), and signal transducer and activator of transcription (STAT) pathways (Figure 3; Goetz and Mohammadi, 2013; Ornitz and Itoh, 2015).

### Development

The definitive endoderm, one of the embryonic germ layers, produces the gut tube and associated organs including the liver, lungs, and pancreas. Liver development, which is initiated by liver budding, occurs through reciprocal inductive interactions between the endoderm and underlying mesoderm. Secreted signals from the mesoderm to the endoderm are essential for liver budding. These secreted signals include FGFs, bone morphogenetic proteins (BMPs), and Wnts (Calmont et al., 2006; Tremblay, 2010). FGF8 and FGF10 play roles as paracrine signals in liver development (Table 1).

**Table 1:** **Roles of paracrine/endocrine FGFs in the liver**.

**FGFs**	**Roles**	**Species**
**PARACRINE FGFs**		
FGF8	A morphogen in liver development	Mice
FGF10	A morphogen in liver development	Mice
FGF7	A repair factor for liver injury	Mice/Humans
FGF9	A repair factor for liver injury	Mice
FGF5	A protector for NASH induced by a high-fat diet	Mice
FGF8	A progressor in HCC	Humans
FGF17	A progressor in HCC	Humans
FGF18	A progressor in HCC	Humans
FGF5	A target of miRNA in HCC	Humans
FGF9	A target of miRNA in HCC	Humans
**ENDOCRINE FGFs**		
FGF15/19	An ilium-derived regulator in hepatic bile acid, protein, and, glycogen metabolism	Mice
FGF21	A liver-derived regulator in glucose and lipid metabolism	Mice
FGF21	A liver-derived protector for NAFLD/NASH	Mice/Humans
FGF15/19	An ilium-derived factor in liver regeneration	Mice
FGF15/19	An ilium-derived progressor in HCC	Mice/Humans

### FGF8 as a morphogen

FGF8 mainly activates FGFR1c with heparan sulfate as a co-factor. *FGF8* is expressed in the adjacent mesoderm in the early embryonic stages, indicating its involvement in liver development (Crossley and Martin, 1995; Calmont et al., 2006; Wang et al., 2015). Since *FGF8* knockout mice, which are lethal at the gastrulation stage, lack all embryonic mesoderm and endoderm-derived structures, the examination of its roles in liver development has been precluded (Sun et al., 1999). However, FGF8 is required for anterior heart field development; therefore, FGF8 is also expected to be required for liver development (Table 1; Ilagan et al., 2006).

### FGF10 as a morphogen

FGF10 preferentially activates FGFR2b with heparan sulfate as a co-factor. FGF10 is also expressed in the adjacent mesoderm in the early embryonic stages, indicating its involvement in liver development (Kelly et al., 2001). *FGF10* knockout mice are lethal shortly after birth due to the lack of multiple organs including limbs and lungs (Sekine et al., 1999). In addition, *FGF10* knockout mice with smaller livers exhibit the reduced proliferation and survival of hepatoblasts, indicating that FGF10 is required for liver growth during embryogenesis and hepatoblast growth (Table 1; Berg et al., 2007).

### Repair

The liver mainly comprises two types of cells: parenchymal (hepatocytes) and non-parenchymal cells. Hepatocytes, which account for ~80% of hepatic cells, perform most metabolic functions in the liver. Hepatocytes also maintain the ability to proliferate in response to toxic injury and infection (Taub, 2004). FGF7 and FGF9 play roles as paracrine signals in liver repair (Table 1).

### FGF7 as a repair factor

FGF7 preferentially activates FGFR2b with heparan sulfate as a co-factor. *FGF7* is a paracrine *FGF* expressed in multiple tissues including the cerebrum, lungs, vas deferens, tongue, and skin in the postnatal stages (Fon Tacer et al., 2010). When hepatocyte proliferation is impaired by severe liver damage, facultative liver progenitor cells proliferate and contribute to repair. The expansion of liver progenitor cells is also often observed in patients with liver diseases. *FGF7* knockout mice, which are viable, have impaired hair, kidney, and neuronal synapse development, but not impaired liver development (Guo et al., 1996; Qiao et al., 1999; Terauchi et al., 2010). However, liver progenitor cell expansion and higher mortality upon toxin-induced hepatic injury are markedly depressed in *FGF7* knockout mice. In contrast, liver progenitor cells are induced and hepatic dysfunction is ameliorated in *FGF7* transgenic mice. The expression of *FGF7* is also induced concomitantly with liver progenitor cell responses in the livers of mouse models as well as in the serum of patients with acute liver failure. These findings indicate that FGF7 is a critical regulator of liver progenitor cells in liver injury (Table 1; Takase et al., 2013).

### FGF9 as a repair factor

FGF9 mainly activates FGFR1c with heparan sulfate as a co-factor. *FGF9* is broadly expressed at high levels throughout the brain and in the kidney in the postnatal stages (Fon Tacer et al., 2010). *FGF9* knockout mice are lethal at the neonatal stage due to lung hypoplasia, but do not have impaired livers (Colvin et al., 2001). Hepatic injury is associated with the activation of hepatic stellate cells. The expression of *FGF9* is increased in hepatic stellate cells in liver slice cultures after exposure to carbon tetrachloride as an acute liver injury model. FGF9 significantly stimulates the incorporation of thymidine by hepatocytes. These findings indicate that FGF9 provides a paracrine mitogenic signal to hepatocytes during acute liver injury (Table 1; Antoine et al., 2007).

### Non-alcoholic fatty liver disease (NAFLD)

NAFLD includes metabolic liver disorders ranging from simple fatty liver (hepatic steatosis) to non-alcoholic steatohepatitis (NASH) and liver cirrhosis. NAFLD, which has high morbidity and mortality rates, is regarded as a serious public health issue. The main risk factors for NAFLD are obesity, dyslipidemia, and insulin resistance. A multi-hit process including lipotoxicity, oxidative stress, endoplasmic reticulum stress, and an inflammatory state has been implicated in the pathogenesis of NAFLD (Takaki et al., 2013; Demir et al., 2015). FGF5 functions as a paracrine signal in NASH (Table 1).

### FGF5 knockout mice fed a high-fat diet with the characteristics of NASH

*FGF5* is expressed throughout the central nervous system and is also present in the skin in the postnatal stages (Fon Tacer et al., 2010). *FGF5* knockout mice with the long hair phenotype are viable and appear to be healthy (Hébert et al., 1994). However, *FGF5* knockout mice fed a high-fat diet gain little weight and have higher serum alanine transaminase, aspartate amino transferase, and non-high-density lipoprotein-cholesterol levels. Their liver histology indicates marked inflammation, focal necrosis, fat deposition, and fibrosis, which are similar to the characteristics of NASH. However, the mechanisms underlying the associations between FGF5, high-fat diet, and NASH currently remain unclear (Table 1; Hanaka et al., 2014).

### Hepatocellular carcinoma (HCC)

HCC is the most common type of liver cancer and has a poor prognosis. Most cases of HCC are secondary to a viral hepatitis infection or cirrhosis. Hepatocarcinogenesis in cirrhosis involves multiple processes, in which precancerous dysplastic nodules transform into early HCC, progressed HCC, and advanced HCC (Forner et al., 2012; Schulze et al., 2015). FGF signaling plays crucial roles in HCC. FGFR3 and FGFR4, which are the main FGFRs expressed in the liver, are involved in HCC (Qiu et al., 2005; French et al., 2012). FGFR2 has also been shown to play a role in HCC (Harimoto et al., 2010). In addition, FGF8, FGF17, and FGF18 act as paracrine signals in HCC. FGF5 and FGF9 are also targets of miRNAs in HCC (Table 1).

### FGF8, FGF17, and FGF18 as progressors

FGF8, FGF17, and FGF18 are members of the FGF8/7/18 subfamily (Figure 1). These FGFs mainly activate FGFR1c with heparan sulfate as a co-factor. At least one member of the FGF8/17/18 subfamily is up-regulated in 59% of 34 human HCC cases. The expression of subfamily members is markedly increased in cultured HCC cells subjected to serum withdrawal or a hypoxia-mimetic drug. The addition of FGF8, FGF17, or FGF18 impairs apoptosis, the incidence of which is elevated in serum-starved cells. In contrast, the down-regulation of *FGF18* by small interfering RNA significantly reduces the viability of HCC cells. FGF8, FGF17, and FGF18 are involved in autocrine/paracrine signaling in HCC and enhance the survival of tumor cells. These findings indicate that FGF8 subfamily members support the development and progression of hepatocellular malignancy (Table 1; Gauglhofer et al., 2011).

### FGF5 and FGF9 as targets of microRNA (miRNAs)

miRNAs are highly conserved small non-coding regulatory RNAs that negatively regulate gene expression by binding directly to corresponding target mRNAs in a sequence-specific manner (Giordano and Columbano, 2013). *miR-188-5p* is significantly decreased in HCC cells and strongly correlates with multiple nodules, microvascular invasion, and the overall and disease-free survival of HCC. The ectopic expression of *miR-188-5p* suppresses HCC cell proliferation and metastasis. The enforced expression of *miR-188-5p* significantly inhibits the expression of *FGF5*, while the restoration of *FGF5* expression reverses the inhibitory effects of *miR-188-5p* on HCC cell proliferation and metastasis. These findings indicate a tumor suppressor role for *miR-188-5p* via the targeting of FGF5, which mainly activates FGFR1c with heparan sulfate as a co-factor in human HCC (Table 1; Fang et al., 2015). Furthermore, *miR-140-5p* suppresses tumor growth and metastasis by targeting FGF9, which also mainly activates FGFR1c with heparan sulfate as a co-factor, in human HCC (Table 1; Kuro-o, 2012; Yang et al., 2013).

## Roles of endocrine FGFs in the liver

Endocrine FGFs, which comprise three members: FGF15/19, FGF21, and FGF23, have a secreted signal sequence and Klotho-binding site at their amino and carboxyl termini (Figure 2A). In contrast to paracrine FGFs, endocrine FGFs do not function as local signals due to their lower heparan sulfate-binding affinity. They require αKlotho or βKlotho as a co-factor for FGFR. αKlotho and βKlotho are specifically expressed in the target tissues of endocrine FGFs, which function in an endocrine manner with target-tissue specificity through the bloodstream (Figure 2B; Goetz and Mohammadi, 2013; Ornitz and Itoh, 2015).

αKlotho and βKlotho, which share structural similarities and characteristics with each other, are single-pass transmembrane proteins of ~1000 amino acids with a short cytoplasmic domain. However, endocrine FGFs cannot efficiently bind to FGFR, αKlotho, or βKlotho alone; they efficiently bind to the FGFR-Klotho complex. FGF15/19 activates FGFR4 with βKlotho and FGF21 activates FGFR1c with βKlotho, which, in turn, induces the activation of intracellular signaling pathways (Figure 3; Beenken and Mohammadi, 2012; Kuro-o, 2012). FGF15/19 exhibits metabolic and proliferative activities. However, FGF21 is a unique FGF with metabolic, but no proliferative activity (Goetz and Mohammadi, 2013; Ornitz and Itoh, 2015).

### Metabolism

The liver plays important roles in multiple catabolic and anabolic pathways including bile acids, lipids, and carbohydrates, in addition to detoxification (Taub, 2004; Bhatia et al., 2014). FGF15/19 acts as an endocrine signal in bile acid metabolism in the liver. FGF21 also functions as an endocrine hepatokine in glucose and lipid metabolism (Table 1).

### FGF15/19 as a regulator of bile acid metabolism

Most *FGF15/19* knockout mice gradually die after embryonic day (E) 10.5 due to defects in the cardiac outflow tract, indicating that FGF15/19 plays a crucial role in embryonic heart development (McWhirter et al., 1997; Vincentz et al., 2005). However, few *FGF15/19* knockout mice survive, even after the postnatal stages. In the postnatal stages, *FGF15/19* is preferentially expressed in the ileum. Bile acids, which are synthesized in the liver, promote the digestion and absorption of dietary fat by forming micelles. The liver and intestines play crucial roles in maintaining bile acid homeostasis. Cholesterol 7α-hydroxylase (CYP7A1) catalyzes the first and rate-limiting step in the bile acid synthetic pathway in the liver. The expression of *FGF15/19* in the ileum is induced by the nuclear bile acid receptor FXR. The induction of *FGF15/19* represses *CYP7A1* expression in the liver in an endocrine manner. Surviving *FGF15/19* knockout mice have higher *CYP7A1* expression levels and enhanced fecal bile acid excretion, indicating that FGF15/19 plays a crucial role in bile acid synthesis as a gut-liver signal in an endocrine manner (Table 1; Inagaki et al., 2005). The cycle of gallbladder filling and emptying controls the flow of bile into the intestines for digestion. Gallbladders in surviving *FGF15/19* knockout mice are almost devoid of bile. The gallbladder volume in surviving *FGF15/19* knockout mice is significantly increased by an injection of the FGF15/19 protein. These findings indicate that FGF15/19 also plays crucial roles in gallbladder filling (Table 1; Choi et al., 2006). FGF15/19 stimulates hepatic protein and glycogen synthesis, but does not induce lipogenesis. Surviving *FGF15/19* knockout mice fail to properly maintain blood concentrations of glucose and normal postprandial amounts of liver glycogen. FGF15/19 treatments restore the loss of glycogen in streptozotocin-treated mice that are diabetic and have no detectable blood insulin levels. These findings indicate that FGF15/19 is a postprandial, insulin-independent activator that acts directly on the liver via hepatic protein and glycogen synthesis (Table 1; Kir et al., 2011).

### FGF21 as a hepatokine in glucose and lipid metabolism

Liver-derived cytokines are known to function as hepatokines and include angiopoietin-related protein 6, fetuin-A, insulin-like growth factors, and selenoprotein P. Hepatokines directly regulate glucose and lipid metabolism (Stefan and Häring, 2013). *FGF21*, which is abundantly expressed in the liver, also plays roles as a hepatokine in glucose and lipid metabolism in white adipose tissue (Itoh, 2014). Although hepatic *FGF21* is generally expressed at low levels, its expression is strongly induced during fasting through the activation of peroxisome proliferator-activated receptor α (PPARα) by the non-esterified fatty acids released from adipocytes and taken up by hepatocytes (Murata et al., 2011). *FGF21* knockout mice are viable, fertile, and appear to be normal. Lipolysis in white adipose tissue is enhanced in fasted *Fgf21* knockout mice, indicating that FGF21 inhibits lipolysis during fasting (Hotta et al., 2009). Hepatic *Fgf21* expression is also significantly induced by a low-carbohydrate, high-fat ketogenic diet. Insulin sensitivity in white adipose tissue is impaired by ketogenic diet feeding. This impaired sensitivity is improved in *FGF21* knockout mice, indicating that FGF21 is a negative regulator of adipocyte insulin sensitivity in adaptation to a low-carbohydrate malnutritional state (Murata et al., 2013). Hepatic *FGF21* induced by starvation also increases systemic glucocorticoid levels and suppresses physical activity in adaptation to starvation responses. These effects in mice were not observed in perfused livers or cultured hepatocytes (Ogawa et al., 2007; Potthoff et al., 2009). In addition, most of these effects have not been observed in mice lacking β*-Klotho* in the brain or those lacking *FGFR1* in adipose tissue (Adams et al., 2010; Owen et al., 2014). These findings indicate that hepatic FGF21 exerts diverse actions through FGFR1c/β-Klotho in the brain or adipose tissue, but not directly on the liver. Hepatic *FGF21* expression is also significantly induced by different kinds of stresses such as hepatic injury, chemical insults, and diseases, indicating that hepatic FGF21 is a stress-induced metabolic regulator (Table 1; Cheng et al., 2014).

Adiponectin is a white adipocyte-derived hormone. Studies on *FGF21* knockout mice have also indicated that hepatic FGF21 is an upstream effector of adiponectin that mediates many of the systemic effects of FGF21 on energy metabolism and insulin sensitivity in the liver and skeletal muscle (Holland et al., 2013; Lin et al., 2013). Glucagon regulates glucose and lipid metabolism and promotes weight loss. Glucagon receptor activation increases hepatic *FGF21* expression. Findings obtained from *FGF21* knockout mice suggest that hepatic FGF21 contributes, at least in part, to glucose, energy, and lipid metabolism controlled by glucagon (Habegger et al., 2013). *FGF21* knockout mice also exhibit insulin resistance while being normoglycemic, and this is associated with increases in pancreatic beta-cell proliferation and insulin synthesis, which act as compensatory responses. This resistance results from enhanced growth hormone sensitivity in *FGF21* knockout pancreatic islets, indicating that hepatic FGF21 is important for the regulation of pancreatic beta-cell proliferation and insulin synthesis, possibly via the modulation of growth hormone signaling (So et al., 2015). Endoplasmic reticulum (ER) stress leads to the development and progression of various diseases such as obesity and diabetes. ER stress induces hepatic *FGF21* expression. ER stress and the hepatic accumulation of lipids are enhanced in *FGF21* knockout mice, indicating that FGF21 plays a role in adaptive responses to ER stress (Table 1; Kim et al., 2015).

## NAFLD

As described above, NAFLD includes metabolic liver disorders ranging from hepatic steatosis to NASH and liver cirrhosis. FGF21 plays roles in NAFLD (Table 1).

### FGF21 as a protective factor for NAFLD

Serum FGF21 levels are significantly increased in patients with hepatic steatosis and NASH in a manner that is dependent on the degree of steatosis (Yilmaz and Eren, 2012; Shen et al., 2012, 2013; Li et al., 2013; Liu et al., 2015). Mice fed a high-fat diet or methionine/choline-deficient diet are model mice for NASH. Serum FGF21 levels and hepatic *FGF21* expression levels are also significantly higher in these mice (Xu et al., 2009; Tanaka et al., 2015). As described above, FGF21 is an important metabolic regulator of glucose and lipid metabolism. The administration of a high dose of intravenous FGF21 reverses hepatic steatosis, improves insulin sensitivity, and decreases serum glucose levels in mice with NAFLD (Xu et al., 2009). These findings indicate that elevated serum FGF21 levels may be a protective response against glucose-lipid metabolism disorders in patients and mice with NAFLD/NASH (Table 1).

Metformin decreases hepatic gluconeogenesis and increases hepatic fatty acid β-oxidation by activating AMPK (Doycheva and Loomba, 2014). In cultured hepatocytes, metformin stimulates the expression of *FGF21*, which is inhibited by an AMPK inhibitor, indicating that FGF21 may contribute to the therapeutic effects of metformin on NAFLD (Nygaard et al., 2012). However, metformin is not beneficial for patients with NAFLD (Mazza et al., 2012). The nicotinamide adenine dinucleotide-dependent deacetylase sirtuin 1 (SIRT1) tightly regulates fatty acid metabolism in the liver. Prolonged fasting induces lipid deposition in the livers of wild-type mice, but severe hepatic steatosis in liver-specific *SIRT1* knockout mice. Fasting increases the expression of *FGF21* in the livers of wild-type mice, but not in those of liver-specific *SIRT1* knockout mice. Decreased hepatic *FGF21* expression and serum FGF21 levels in fasted liver-specific *SIRT1* knockout mice have been correlated with the decreased hepatic expression of genes involved in fatty acid oxidation and ketogenesis, and increased expression of genes that control lipogenesis. The SIRT1 activator, resveratrol, increases *FGF21* mRNA and FGF21 protein levels in human liver carcinoma HepG2 cells. The hepatic overexpression of *FGF21* in liver-specific *SIRT1* knockout mice increases the expression of genes involved in fatty acid oxidation, thereby decreasing fasting-induced steatosis, reducing obesity, increasing energy expenditure, and promoting the browning of white adipose tissue. These findings indicate that the SIRT1-mediated activation of *FGF21* prevents liver steatosis caused by fasting (Table 1; Li et al., 2014).

### Regeneration

Hepatocytes, which are long lived, normally do not undergo cell division. After the surgical removal of two-thirds of the liver in mice, the remaining liver enlarges until the original liver is restored. The liver has a strong regenerative capacity due to hyperplastic responses that involve the replication of all mature functioning cells in the remnant liver without the recruitment of liver stem cells or progenitor cells (Taub, 2004). FGF15/19 functions as an endocrine signal in liver regeneration (Table 1).

### FGF15/19 as a regenerative factor

As described above, *FGF15/19* is preferentially expressed in the ileum. After 2/3 partial hepatectomy, *FGF15/19* knockout mice display more extensive liver necrosis and greater elevations in serum bile acid and bilirubin levels than wild-type mice. In addition, hepatocyte proliferation is reduced in *FGF15/19* knockout mice because of impaired cell cycle progression, indicating that FGF15/19 is required for liver regeneration. The underlying mechanisms are likely the result of disrupted bile acid homeostasis and the impaired priming of hepatocyte proliferation (Table 1; Uriarte et al., 2013; Kong et al., 2014).

## HCC

As described above, HCC is the most common type of liver cancer, in which FGF15/19 plays roles (Table 1).

### FGF15/19 as a progressor

Fewer and smaller tumors with smaller histological neoplastic lesions are observed in *FGF15/19* knockout mice subjected to a clinically relevant model of liver inflammation and fibrosis-associated carcinogenesis. Ileal *FGF15/19* expression is stimulated in mice undergoing carcinogenesis. Hepatocellular proliferation and fibrogenesis are also reduced in *FGF15/19* knockout mice. *In vitro* experiments indicate that liver fibrogenic stellate cells are not direct targets for FGF15/FGF19. FGF15/FGF19 as an endocrine factor induces the expression of pro-fibrogenic and pro-tumorigenic *connective tissue growth factor* (*CTGF*) in hepatocytes. These findings indicate the existence of FGF15/19-triggered CTGF-mediated paracrine effects on stellate cells, and an amplification mechanism for the hepatocarcinogenic effects of FGF15/19 via the production of CTGF (Table 1; Uriarte et al., 2015).

In contrast, *FGF15/19* is significantly overexpressed in human HCC. Serum FGF15/19 levels in patients with HCC are significantly decreased after hepatectomy. The FGF15/19 protein increases the proliferation and inversion capabilities of cultured human hepatocellular carcinoma cells. These findings indicate that FGF15/19 functions as an autocrine/paracrine factor in human HCC (Miura et al., 2012). Epithelial-mesenchymal transition is a key event in metastasis and plays a critical role in the progression of HCC (van Zijl et al., 2009). The expression of *FGF15/19* is significantly elevated and negatively associated with the expression of *E-cadherin* in HCC tissues and cell lines. Ectopic *FGF15/19* expression promotes epithelial-mesenchymal transition and invasion in epithelial-like HCC cells through the repression of *E-cadherin* expression, whereas *FGF15/19* knockdown enhances *E-cadherin* expression and, hence, diminishes epithelial-mesenchymal transition traits in mesenchymal-like HCC cells. However, *FGF15/19* knockdown cannot abrogate epithelial-mesenchymal transition traits in the presence of glycogen synthase kinase 3β (GSK3β) inhibitors. FGF15/19-induced epithelial-mesenchymal transition may be markedly attenuated when *FGFR4* is knocked out. These findings indicate that the FGFR4/GSK3β/β-catenin axis plays a pivotal role in FGF15/19-induced epithelial-mesenchymal transition in HCC cells (Table 1; Zhao et al., 2015).

Genomic analyses promise to improve tumor characterization in order to optimize personalized treatments for patients with HCC. Exome sequencing analyses of HCC indicate that the gene locus including *FGF15/19* is amplified at advanced stages in aggressive HCC (Table 1; Schulze et al., 2015).

## Conclusions

FGFs are growth factors with diverse functions in development, health, and disease. Among 22 FGFs, several FGFs act as paracrine or endocrine signals in liver development, health, and disease. FGF8 and FGF10 function as paracrine signals in embryonic liver development. FGF7 and FGF9 also act as paracrine signals in repair in response to liver injury. In addition, FGF5, FGF8, FGF9, FGF17, and FGF18 play roles as paracrine signals in the development and progression of hepatocellular carcinoma. In contrast, FGF15/19 and FGF21 are endocrine signals. FGF15/19, which is produced in the ileum, is involved in bile acid metabolism and gallbladder filling in the liver. FGF15/19 is also a postprandial, insulin-independent activator of hepatic protein and glycogen synthesis. Furthermore, FGF15/19 is involved in liver regeneration and hepatocellular carcinoma. FGF21 is a hepatokine produced in the liver. It regulates glucose and lipid metabolism. Serum FGF21 levels are increased in NAFLD. FGF21 also protects against NAFLD. These findings provide new insights into the roles of FGFs in the liver and potential therapeutic strategies for hepatic disorders.

## Author contributions

All authors listed, have made substantial, direct and intellectual contribution to the work, and approved it for publication.

### Conflict of interest statement

The authors declare that the research was conducted in the absence of any commercial or financial relationships that could be construed as a potential conflict of interest.

## References

[B1] Adams A. C. Yang C. Coskun T. Cheng C. C. Gimeno R. E. Luo Y.. (2010). The breadth of FGF21's metabolic actions are governed by FGFR1 in adipose tissue. Mol. Metab. 2, 31–37. 10.1016/j.molmet.2012.08.007 24024127 PMC3757657

[B2] Antoine M. Wirz W. Tag C. G. Gressner A. M. Marvituna M. Wycislo M.. (2007). Expression and function of fibroblast growth factor (FGF) 9 in hepatic stellate cells and its role in toxic liver injury. Biochem. Biophys. Res. Commun. 361, 335–341. 10.1016/j.bbrc.2007.06.189 17662249

[B3] Baird A. Klagsbrun M. (1991). The fibroblast growth factor family. Cancer Cells 3, 239–243. 1911037

[B4] Beenken A. Mohammadi M. (2012). The structural biology of the FGF19 subfamily. Adv. Exp. Med. Biol. 728, 1–24. 10.1007/978-1-4614-0887-1_1 22396159 PMC3682411

[B5] Berg T. Rountree C. B. Lee L. Estrada J. Sala F. G. Choe A.. (2007). Fibroblast growth factor 10 is critical for liver growth during embryogenesis and controls hepatoblast survival via beta-catenin activation. Hepatology 46, 1187–1197. 10.1002/hep.21814 17668871 PMC3494299

[B6] Bhatia S. N. Underhill G. H. Zaret K. S. Fox I. J. (2014). Cell and tissue engineering for liver disease. Sci. Transl. Med. 6, 245sr2. 10.1126/scitranslmed.3005975 25031271 PMC4374645

[B7] Burgess W. H. Maciag T. (1989). The heparin-binding (fibroblast) growth factor family of proteins. Annu. Rev. Biochem. 58, 575–606. 10.1146/annurev.bi.58.070189.003043 2549857

[B8] Calmont A. Wandzioch E. Tremblay K. D. Minowada G. Kaestner K. H. Martin G. R.. (2006). An FGF response pathway that mediates hepatic gene induction in embryonic endoderm cells. Dev. Cell 11, 339–348. 10.1016/j.devcel.2006.06.015 16950125

[B9] Cheng X. Vispute S. G. Liu J. Cheng C. Kharitonenkov A. Klaassen C. D. (2014). Fibroblast growth factor (Fgf) 21 is a novel target gene of the aryl hydrocarbon receptor (AhR). Toxicol. Appl. Pharmacol. 278, 65–71. 10.1016/j.taap.2014.04.013 24769090 PMC4090247

[B10] Choi M. Moschetta A. Bookout A. L. Peng L. Umetani M. Holmstrom S. R.. (2006). Identification of a hormonal basis for gallbladder filling. Nat. Med. 12, 1253–12555. 10.1038/nm1501 17072310

[B11] Colvin J. S. White A. C. Pratt S. J. Ornitz D. M. (2001). Lung hypoplasia and neonatal death in Fgf9-null mice identify this gene as an essential regulator of lung mesenchyme. Development 128, 2095–2106. 11493531 10.1242/dev.128.11.2095

[B12] Crossley P. H. Martin G. R. (1995). The mouse Fgf8 gene encodes a family of polypeptides and is expressed in regions that direct outgrowth and patterning in the developing embryo. Development 121, 439–451. 7768185 10.1242/dev.121.2.439

[B13] Demir M. Lang S. Steffen H. M. (2015). Nonalcoholic fatty liver disease: current status and future directions. J. Dig. Dis. 16, 541–557. 10.1111/1751-2980.12291 26406351

[B14] Doycheva I. Loomba R. (2014). Effect of metformin on ballooning degeneration in nonalcoholic steatohepatitis (NASH): when to use metformin in nonalcoholic fatty liver disease (NAFLD). Adv. Ther. 31, 30–43. 10.1007/s12325-013-0084-6 24385405

[B15] Fang F. Chang R. M. Yu L. Lei X. Xiao S. Yang H.. (2015). MicroRNA-188-5p suppresses tumor cell proliferation and metastasis by directly targeting FGF5 in hepatocellular carcinoma. J. Hepatol. 63, 874–885. 10.1016/j.jhep.2015.05.008 25998163

[B16] Fon Tacer K. Bookout A. L. Ding X. Kurosu H. John G. B. Wang L.. (2010). Research resource: comprehensive expression atlas of the fibroblast growth factor system in adult mouse. Mol. Endocrinol. 24, 2050–2064. 10.1210/me.2010-0142 20667984 PMC2954642

[B17] Forner A. Llovet J. M. Bruix J. (2012). Chemoembolization for intermediate HCC: is there proof of survival benefit? J. Hepatol. 56, 984–986. 10.1016/j.jhep.2011.08.017 22008737

[B18] French D. M. Lin B. C. Wang M. Adams C. Shek T. Hötzel K.. (2012). Targeting FGFR4 inhibits hepatocellular carcinoma in preclinical mouse models. PLoS ONE 7:e36713. 10.1371/journal.pone.0036713 22615798 PMC3352934

[B19] Gauglhofer C. Sagmeister S. Schrottmaier W. Fischer C. Rodgarkia-Dara C. Mohr T.. (2011). Up-regulation of the fibroblast growth factor 8 subfamily in human hepatocellular carcinoma for cell survival and neoangiogenesis. Hepatology 53, 854–864. 10.1002/hep.24099 21319186

[B20] Giordano S. Columbano A. (2013). MicroRNAs: new tools for diagnosis, prognosis, and therapy in hepatocellular carcinoma? Hepatology 57, 840–847. 10.1002/hep.26095 23081718

[B21] Goetz R. Mohammadi M. (2013). Exploring mechanisms of FGF signalling through the lens of structural biology. Nat. Rev. Mol. Cell. Biol. 14, 166–180. 10.1038/nrm3528 23403721 PMC3695728

[B22] Guo L. Degenstein L. Fuchs E. (1996). Keratinocyte growth factor is required for hair development but not for wound healing. Genes Dev. 10, 165–175. 10.1101/gad.10.2.165 8566750

[B23] Habegger K. M. Stemmer K. Cheng C. Müller T. D. Heppner K. M. Ottaway N.. (2013). Fibroblast growth factor 21 mediates specific glucagon actions. Diabetes 62, 1453–1463. 10.2337/db12-1116 23305646 PMC3636653

[B24] Hanaka H. Hamada T. Ito M. Nakashima H. Tomita K. Seki S.. (2014). Fibroblast growth factor-5 participates in the progression of hepatic fibrosis. Exp. Anim. 63, 85–92. 10.1538/expanim.63.85 24521867 PMC4160928

[B25] Harimoto N. Taguchi K. Shirabe K. Adachi E. Sakaguchi Y. Toh Y.. (2010). The significance of fibroblast growth factor receptor 2 expression in differentiation of hepatocellular carcinoma. Oncology 78, 361–368. 10.1159/000320463 20798558

[B26] Hébert J. M. Rosenquist T. Götz J. Martin G. R. (1994). FGF5 as a regulator of the hair growth cycle: evidence from targeted and spontaneous mutations. Cell 78, 1017–1025. 10.1016/0092-8674(94)90276-3 7923352

[B27] Holland W. L. Adams A. C. Brozinick J. T. Bui H. H. Miyauchi Y. Kusminski C. M.. (2013). An FGF21-adiponectin-ceramide axis controls energy expenditure and insulin action in mice. Cell Metab. 17, 790–797. 10.1016/j.cmet.2013.03.019 23663742 PMC3667496

[B28] Hotta Y. Nakamura H. Konishi M. Murata Y. Takagi H. Matsumura S. (2009). Fibroblast growth factor 21 regulates lipolysis in white adipose tissue but is not required for ketogenesis and triglyceride clearance in liver. Endocrinology 150, 4625–4633. 10.1210/en.2009-0119 19589869

[B29] Ilagan R. Abu-Issa R. Brown D. Yang Y. P. Jiao K. Schwartz R. J.. (2006). Fgf8 is required for anterior heart field development. Development 133, 2435–2445. 10.1242/dev.02408 16720880

[B30] Inagaki T. Choi M. Moschetta A. Peng L. Cummins C. L. McDonald J. G.. (2005). Fibroblast growth factor 15 functions as an enterohepatic signal to regulate bile acid homeostasis. Cell Metab. 2, 217–225. 10.1016/j.cmet.2005.09.001 16213224

[B31] Itoh N. (2014). FGF21 as a hepatokine, adipokine, and myokine in metabolism and diseases. Front. Endocrinol. 5:107. 10.3389/fendo.2014.00107 25071723 PMC4083219

[B32] Kelly R. G. Brown N. A. Buckingham M. E. (2001). The arterial pole of the mouse heart forms from Fgf10-expressing cells in pharyngeal mesoderm. Dev. Cell 1, 435–440. 10.1016/S1534-5807(01)00040-5 11702954

[B33] Kim S. H. Kim K. H. Kim H. K. Kim M. J. Back S. H. Konishi M.. (2015). Fibroblast growth factor 21 participates in adaptation to endoplasmic reticulum stress and attenuates obesity-induced hepatic metabolic stress. Diabetologia 58, 809–818. 10.1007/s00125-014-3475-6 25537833

[B34] Kir S. Beddow S. A. Samuel V. T. Miller P. Previs S. F. Suino-Powell K.. (2011). FGF19 as a postprandial, insulin-independent activator of hepatic protein and glycogen synthesis. Science 331, 1621–1624. 10.1126/science.1198363 21436455 PMC3076083

[B35] Kong B. Huang J. Zhu Y. Li G. Williams J. Shen S.. (2014). Fibroblast growth factor 15 deficiency impairs liver regeneration in mice. Am. J. Physiol. Gastrointest. Liver Physiol. 306, G893–G902. 10.1152/ajpgi.00337.2013 24699334 PMC4024724

[B36] Kuro-o M. (2012). Klotho and βKlotho. Adv. Exp. Med. Biol. 728, 25–40. 10.1007/978-1-4614-0887-1_2 22396160

[B37] Li H. Dong K. Fang Q. Hou X. Zhou M. Bao Y.. (2013). High serum level of fibroblast growth factor 21 is an independent predictor of non-alcoholic fatty liver disease: a 3-year prospective study in China. J. Hepatol. 58, 557–563. 10.1016/j.jhep.2012.10.029 23142063

[B38] Li Y. Wong K. Giles A. Jiang J. Lee J. W. Adams A. C.. (2014). Hepatic SIRT1 attenuates hepatic steatosis and controls energy balance in mice by inducing fibroblast growth factor 21. Gastroenterology 146, 539.e7–549.e7. 10.1053/j.gastro.2013.10.059 24184811 PMC4228483

[B39] Lin Z. Tian H. Lam K. S. Lin S. Hoo R. C. Konishi M.. (2013). Adiponectin mediates the metabolic effects of FGF21 on glucose homeostasis and insulin sensitivity in mice. Cell Metab. 17, 779–789. 10.1016/j.cmet.2013.04.005 23663741

[B40] Liu J. Xu Y. Hu Y. Wang G. (2015). The role of fibroblast growth factor 21 in the pathogenesis of non-alcoholic fatty liver disease and implications for therapy. Metabolism 64, 380–390. 10.1016/j.metabol.2014.11.009 25516477

[B41] Mazza A. Fruci B. Garinis G. A. Giuliano S. Malaguarnera R. Belfiore A. (2012). The role of metformin in the management of NAFLD. Exp. Diabetes Res. 2012:716404. 10.1155/2012/716404 22194737 PMC3238361

[B42] McWhirter J. R. Goulding M. Weiner J. A. Chun J. Murre C. (1997). A novel fibroblast growth factor gene expressed in the developing nervous system is a downstream target of the chimeric homeodomain oncoprotein E2A-Pbx1. Development 124, 3221–3232. 9310317 10.1242/dev.124.17.3221

[B43] Miura S. Mitsuhashi N. Shimizu H. Kimura F. Yoshidome H. Otsuka M.. (2012). Fibroblast growth factor 19 expression correlates with tumor progression and poorer prognosis of hepatocellular carcinoma. BMC Cancer 12:56. 10.1186/1471-2407-12-56 22309595 PMC3293719

[B44] Murata Y. Konishi M. Itoh N. (2011). FGF21 as an endocrine regulator in lipid metabolism: from molecular evolution to physiology and pathophysiology. J. Nutr. Metab. 2011:981315. 10.1155/2011/981315 21331285 PMC3038562

[B45] Murata Y. Nishio K. Mochiyama T. Konishi M. Shimada M. Ohta H.. (2013). Fgf21 impairs adipocyte insulin sensitivity in mice fed a low-carbohydrate, high-fat ketogenic diet. PLoS ONE 8:e69330. 10.1371/journal.pone.0069330 23874946 PMC3706421

[B46] Nygaard E. B. Vienberg S. G. Ørskov C. Hansen H. S. Andersen B. (2012). Metformin stimulates FGF21 expression in primary hepatocytes. Exp. Diabetes Res. 2012:465282. 10.1155/2012/465282 23118742 PMC3478742

[B47] Ogawa Y. Kurosu H. Yamamoto M. Nandi A. Rosenblatt K. P. Goetz R.. (2007). BetaKlotho is required for metabolic activity of fibroblast growth factor 21. Proc. Natl. Acad. Sci. U.S.A. 104, 7432–7437. 10.1073/pnas.0701600104 17452648 PMC1855074

[B48] Ornitz D. M. Itoh N. (2015). The fibroblast growth factor signaling pathway. Wiley Interdiscip. Rev. Dev. Biol. 4, 215–266. 10.1002/wdev.176 25772309 PMC4393358

[B49] Owen B. M. Ding X. Morgan D. A. Coate K. C. Bookout A. L. Rahmouni K.. (2014). FGF21 acts centrally to induce sympathetic nerve activity, energy expenditure, and weight loss. Cell Metab. 20, 670–677. 10.1016/j.cmet.2014.07.012 25130400 PMC4192037

[B50] Potthoff M. J. Inagaki T. Satapati S. Ding X. He T. Goetz R.. (2009). FGF21 induces PGC-1alpha and regulates carbohydrate and fatty acid metabolism during the adaptive starvation response. Proc. Natl. Acad. Sci. U.S.A. 106, 10853–10858. 10.1073/pnas.0904187106 19541642 PMC2705613

[B51] Qiao J. Uzzo R. Obara-Ishihara T. Degenstein L. Fuchs E. Herzlinger D. (1999). FGF-7 modulates ureteric bud growth and nephron number in the developing kidney. Development 126, 547–554. 9876183 10.1242/dev.126.3.547

[B52] Qiu W. H. Zhou B. S. Chu P. G. Chen W. G. Chung C. Shih J.. (2005). Over-expression of fibroblast growth factor receptor 3 in human hepatocellular carcinoma. World J. Gastroenterol. 11, 5266–5272. 10.3748/wjg.v11.i34.5266 16149130 PMC4622793

[B53] Schulze K. Imbeaud S. Letouzé E. Alexandrov L. B. Calderaro J. Rebouissou S.. (2015). Exome sequencing of hepatocellular carcinomas identifies new mutational signatures and potential therapeutic targets. Nat. Genet. 47, 505–511. 10.1038/ng.3252 25822088 PMC4587544

[B54] Sekine K. Ohuchi H. Fujiwara M. Yamasaki M. Yoshizawa T. Sato T.. (1999). Fgf10 is essential for limb and lung formation. Nat. Genet. 21, 138–141. 9916808 10.1038/5096

[B55] Shen J. Chan H. L. Wong G. L. Choi P. C. Chan A. W. Chan H. Y.. (2012). Non-invasive diagnosis of non-alcoholic steatohepatitis by combined serum biomarkers. J. Hepatol. 56, 1363–1370. 10.1016/j.jhep.2011.12.025 22314419

[B56] Shen Y. Ma X. Zhou J. Pan X. Hao Y. Zhou M.. (2013). Additive relationship between serum fibroblast growth factor 21 level and coronary artery disease. Cardiovasc. Diabetol. 12:124. 10.1186/1475-2840-12-124 23981342 PMC3766150

[B57] So W. Y. Cheng Q. Xu A. Lam K. S. Leung P. S. (2015). Loss of fibroblast growth factor 21 action induces insulin resistance, pancreatic islet hyperplasia and dysfunction in mice. Cell Death Dis. 6, e1707. 10.1038/cddis.2015.80 25811804 PMC4385948

[B58] Stefan N. Häring H. U. (2013). The role of hepatokines in metabolism. Nat. Rev. Endocrinol. 9, 144–152. 10.1038/nrendo.2012.258 23337953

[B59] Sun X. Meyers E. N. Lewandoski M. Martin G. R. (1999). Targeted disruption of Fgf8 causes failure of cell migration in the gastrulating mouse embryo. Genes Dev. 13, 1834–1846. 10.1101/gad.13.14.1834 10421635 PMC316887

[B60] Takaki A. Kawai D. Yamamoto K. (2013). Multiple hits, including oxidative stress, as pathogenesis and treatment target in non-alcoholic steatohepatitis (NASH). Int. J. Mol. Sci. 14, 20704–20728. 10.3390/ijms141020704 24132155 PMC3821639

[B61] Takase H. M. Itoh T. Ino S. Wang T. Koji T. Akira S.. (2013). FGF7 is a functional niche signal required for stimulation of adult liver progenitor cells that support liver regeneration. Genes Dev. 27, 169–181. 10.1101/gad.204776.112 23322300 PMC3566310

[B62] Tanaka N. Takahashi S. Zhang Y. Krausz K. W. Smith P. B. Patterson A. D.. (2015). Role of fibroblast growth factor 21 in the early stage of NASH induced by methionine- and choline-deficient diet. Biochim. Biophys. Acta 1852, 1242–1252. 10.1016/j.bbadis.2015.02.012 25736301 PMC4433820

[B63] Taub R. (2004). Liver regeneration: from myth to mechanism. Nat. Rev. Mol. Cell Biol. 5, 836–847. 10.1038/nrm1489 15459664

[B64] Terauchi A. Johnson-Venkatesh E. M. Toth A. B. Javed D. Sutton M. A. Umemori H. (2010). Distinct FGFs promote differentiation of excitatory and inhibitory synapses. Nature 465, 783–787. 10.1038/nature09041 20505669 PMC4137042

[B65] Tremblay K. D. (2010). Formation of the murine endoderm: lessons from the mouse, frog, fish, and chick. Prog. Mol. Biol. Transl. Sci. 96, 1–34. 10.1016/B978-0-12-381280-3.00001-4 21075338

[B66] Uriarte I. Fernandez-Barrena M. G. Monte M. J. Latasa M. U. Chang H. C. Carotti S.. (2013). Identification of fibroblast growth factor 15 as a novel mediator of liver regeneration and its application in the prevention of post-resection liver failure in mice. Gut 62, 899–910. 10.1136/gutjnl-2012-302945 23292666

[B67] Uriarte I. Latasa M. U. Carotti S. Fernandez-Barrena M. G. Garcia-Irigoyen O. Elizalde M.. (2015). Ileal FGF15 contributes to fibrosis-associated hepatocellular carcinoma development. Int. J. Cancer 136, 2469–2475. 10.1002/ijc.29287 25346390

[B68] van Zijl F. Zulehner G. Petz M. Schneller D. Kornauth C. Hau M.. (2009). Epithelial-mesenchymal transition in hepatocellular carcinoma. Future Oncol. 5, 1169–1179. 10.2217/fon.09.91 19852728 PMC2963061

[B69] Vincentz J. W. McWhirter J. R. Murre C. Baldini A. Furuta Y. (2005). Fgf15 is required for proper morphogenesis of the mouse cardiac outflow tract. Genesis 41, 192–201. 10.1002/gene.20114 15789410

[B70] Wang J. Rhee S. Palaria A. Tremblay K. D. (2015). FGF signaling is required for anterior but not posterior specification of the murine liver bud. Dev. Dyn. 244, 431–443. 10.1002/dvdy.24215 25302779 PMC4344927

[B71] Xu J. Lloyd D. J. Hale C. Stanislaus S. Chen M. Sivits G.. (2009). Fibroblast growth factor 21 reverses hepatic steatosis, increases energy expenditure, and improves insulin sensitivity in diet-induced obese mice. Diabetes 58, 250–259. 10.2337/db08-0392 18840786 PMC2606881

[B72] Yang H. Fang F. Chang R. Yang L. (2013). MicroRNA-140-5p suppresses tumor growth and metastasis by targeting transforming growth factor β receptor 1 and fibroblast growth factor 9 in hepatocellular carcinoma. Hepatology 58, 205–217. 10.1002/hep.26315 23401231

[B73] Yilmaz Y. Eren F. (2012). Identification of a support vector machine-based biomarker panel with high sensitivity and specificity for nonalcoholic steatohepatitis. Clin. Chim. 414, 154–157. 10.1016/j.cca.2012.08.005 22985537

[B74] Zhao H. Lv F. Liang G. Huang X. Wu G. Zhang W. (2015). FGF19 promotes epithelial-mesenchymal transition in hepatocellular carcinoma cells by modulating the GSK3β/β- catenin signaling cascade via FGFR4 activation. Oncotarget 7, 13575–13586. 10.1186/1471-2407-12-56 PMC4924662 26498355

